# Novel Calcium Zirconate Silicate Cement Biomineralize and Seal Root Canals

**DOI:** 10.3390/ma11040588

**Published:** 2018-04-11

**Authors:** Soram Oh, Sung-In Cho, Hiran Perinpanayagam, Jinsu You, Seong-Hyeon Hong, Yeon-Jee Yoo, Seok Woo Chang, Won-Jun Shon, Jun-Sang Yoo, Seung-Ho Baek, Kee-Yeon Kum

**Affiliations:** 1 Department of Conservative Dentistry, Kyung Hee University Dental Hospital, Kyung Hee University School of Dentistry, 23 Kyungheedae-ro, Dongdaemun-gu, Seoul 02447, Korea; soram0123@gmail.com (S.O.); swc2007smc@gmail.com (S.W.C.); 2 Department of Conservative Dentistry, Dental Research Institute, Seoul National University Dental Hospital & Seoul Dental Hospital for Disabled, Seoul National University School of Dentistry, 101 Daehak-ro, Jongro-gu, Seoul 03080, Korea; gbpaul@naver.com (S.-I.C.); duswl0808@hanmail.net (Y.-J.Y.); endoson@snu.ac.kr (W.-J.S.), shbaek@snu.ac.kr (S.-H.B.); 3 Schulich School of Medicine & Dentistry, University of Western Ontario, 1151 Richmond Street, London, ON N6A3K7, Canada; hperinpa@uwo.ca; 4 Department of Materials Science and Engineering, Seoul National University, 1 Kwanak-ro, Kwanak-gu, Seoul 08826, Korea; jsyou@snu.ac.kr (J.Y.); shhong@snu.ac.kr (S.-H.H.); 5 U Dental Hospital, 175 Dasan-ro, Jung-gu, Seoul 04608, Korea; dryjskr@daum.net

**Keywords:** calcium silicate-based sealer, endotoxin leakage, scanning electron microscopy, sealing ability, calcium zirconate silicate cement

## Abstract

This study evaluated the sealing ability of gutta-percha (GP) with a calcium silicate-based sealer and a novel calcium zirconate containing calcium silicate cement (ZC). The root canals of the extracted premolars were prepared, which were then randomly allocated to three experimental groups (12 root canals per group) for obturation by continuous wave of condensation with the GP and AH 26 sealer (CW); obturation using a single GP with a calcium silicate-based EndoSequence BC sealer (SC); or obturation with ZC. The roots were inserted into sterile Eppendorf tubes, which were inoculated coronally with *Porphyromonas gingivalis*. The amount of endotoxin leakage into the apical reservoirs were measured using the Limulus Amebocyte Lysate (LAL) assay over 21 days, with comparisons made using one-way ANOVA and Scheffe’s tests (α = 0.05). After 21 days, 75% of the canals that had been obturated by SC, 50% of those obturated by CW and 42% of those obturated by ZC showed endotoxin leakage. The amount of leakage was higher in the SC canals than in the CW (*p* = 0.031) or ZC (*p* = 0.03) canals, although there was no significant difference in the amount of leakage for CW and ZC (*p* > 0.05). X-ray diffraction revealed the presence of tricalcium silicate (Ca_3_SiO_5_) and calcium zirconate (CaZrO_3_) in the synthesized ZC. Scanning electron microscopy revealed mineralized precipitates on the dentin of canals obturated by ZC. The novel calcium zirconate silicate cement appears to promote biomineralization and seal root canals at least as effectively as the conventional sealer.

## 1. Introduction

Endodontic treatment needs to eliminate microbes and seal canals [1]. However, the commonly used gutta-percha (GP) cones with root canal sealers do not completely seal root canals [2]. Strategies for enhancing the seal include materials that adhere to dentin [3], self-sealing gaps by setting or hygroscopic expansion of the core material or sealer [4,5], enhancement of the flow and adaptation of fillings within root canal systems [6]. Recently, the materials that form a mineralized layer at the interface with root canal dentin have shown promise in sealing root canals. These include the calcium silicate-based sealers shown to stimulate hydroxyapatite nucleation and bio-mineralization within dentinal tubules, which have been examined in previous studies [7,8,9].

The calcium silicate-based products include Endosequence BC sealer^®^ (EBC, Brasseler, Savannah, GA, USA), which contains Ca_2_SiO_4_, Ca(H_2_PO_4_)_2_, ZrO_2_ and Ca(OH)_2_ [10]. EBC’s post-setting pH of over 10.5 provides antibacterial properties [11,12,13], it releases significantly more calcium ions than the epoxy resin sealer and it can form a hydroxyapatite layer at the interface with dentin [11]. A slight setting expansion (0.087 ± 0.04%) [12] further improves adaptation in the root canal. 

Another calcium silicate filling material is mineral trioxide aggregate (MTA), which provides an excellent seal, is highly biocompatible and promotes biomineralization [14]. Mineralization within the dentinal tubules has been observed in root canals obturated with MTA in vitro [7,15]. However, the bismuth oxide (Bi_2_O_3_) added for radiopacity can retard hydration, prolong the setting time, increase porosity, cause discoloration and may be cytotoxic to human periodontal ligament cells [16,17,18,19]. As substitutes, zirconium oxide (ZrO_2_) had physicochemical properties that were comparable to MTA when ZrO_2_ was used with calcium silicate cement [20,21]. However, ZrO_2_ surfaces may contain heterogeneous nucleation sites for the precipitation and growth of early calcium silicate hydrate gel products [22]. MTA also contains tetracalcium aluminoferrite and some heavy metals in its composition [23,24]. Aluminum is toxic to osteoblasts and inhibits bone mineralization, while ferric ions can discolor teeth [25,26]. Therefore, a novel CaZrO_3_-containing calcium silicate cement (ZC) composed of tricalcium silicate and calcium zirconate was created by a solid-state reaction to exclude ZrO_2_, aluminum and heavy metals.

The aim of this in vitro study was to evaluate the sealing ability of root canals that had been obturated with either the novel ZC, a single GP cone (SC) with EBC or the continuous wave of condensation (CW) using GP and epoxy resin sealer. The endotoxin leakage was measured in canals and biomineralization was identified at the interface with dentin.

## 2. Results

**Figure 1:**
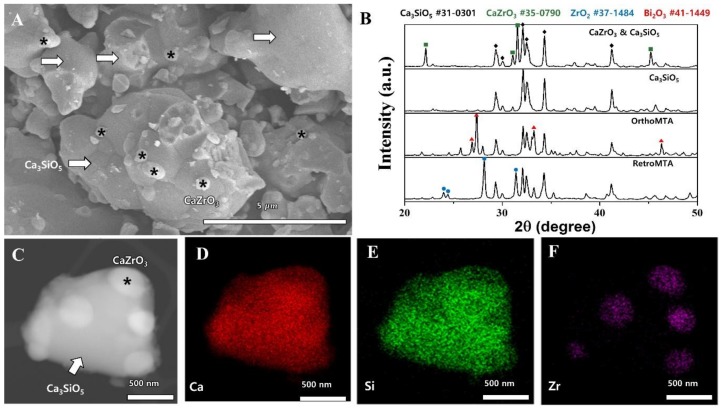
Phase analysis of CaZrO_3_-containing calcium silicate cement. (**A**) FE-SEM image of the synthesized CaZrO_3_-containing calcium silicate cement (×10,000); calcium zirconate (*) was generated, scattered around the tricalcium silicate facet (arrows). (**B**) X-ray diffraction pattern of the synthesized cement, tricalcium silicate without ZrO_2_, OrthoMTA and RetroMTA. The elemental analysis showed the presence of tricalcium silicate (Ca_3_SiO_5_) and calcium zirconate (CaZrO_3_) in the synthesized cement. Bismuth oxide (Bi_2_O_3_) and zirconium oxide (ZrO_2_), which are components of OrthoMTA and RetroMTA, respectively, were not found in the synthesized cement. (**C**) STEM image of the synthesized cement; in which, calcium zirconate (*) and tricalcium silicate (arrow) were observed. (**D**–**F**) EDS mapping of the synthesized cement showed that Zr was locally present on the surface of tricalcium silicate and the small particles on the surface are calcium zirconate (CaZrO_3_).

ZC was synthesized successfully by a solid-state reaction. Field emission scanning electron microscopic (FE-SEM, Model S 4700, Hitachi, Tokyo, Japan) examination of the synthesized cement powders revealed an irregular surface morphology and dimensions of <5 μm. There were small particles <1 μm on the surface of tricalcium silicate (Figure 1A). X-ray diffraction (D8-Advance, Bruker AXS Inc., Madison, WI, USA) (Figure 1B) showed that the synthesized ZC was a mixture of tricalcium silicate (Ca_3_SiO_5_, JCDPS # 31-0301) and calcium zirconate (CaZrO_3_, JCDPS # 35-0790). There was no Bi_2_O_3_ or ZrO_2_, which are found in OrthoMTA (BioMTA, Seoul, Korea) and RetroMTA (BioMTA), respectively (Figure 1B). The scanning transmission electron microscopy (STEM, Model JEM-2100F, JEOL Ltd., Tokyo, Japan) image and energy dispersive spectroscopy (EDS) mappings showed that Ca and Si were uniformly distributed over the entire surface of tricalcium silicate and Zr was locally present (Figure 1C–F). Therefore, the small particles observed on the surface of tricalcium silicate are likely to be calcium zirconate (CaZrO_3_).

**Figure 2:**
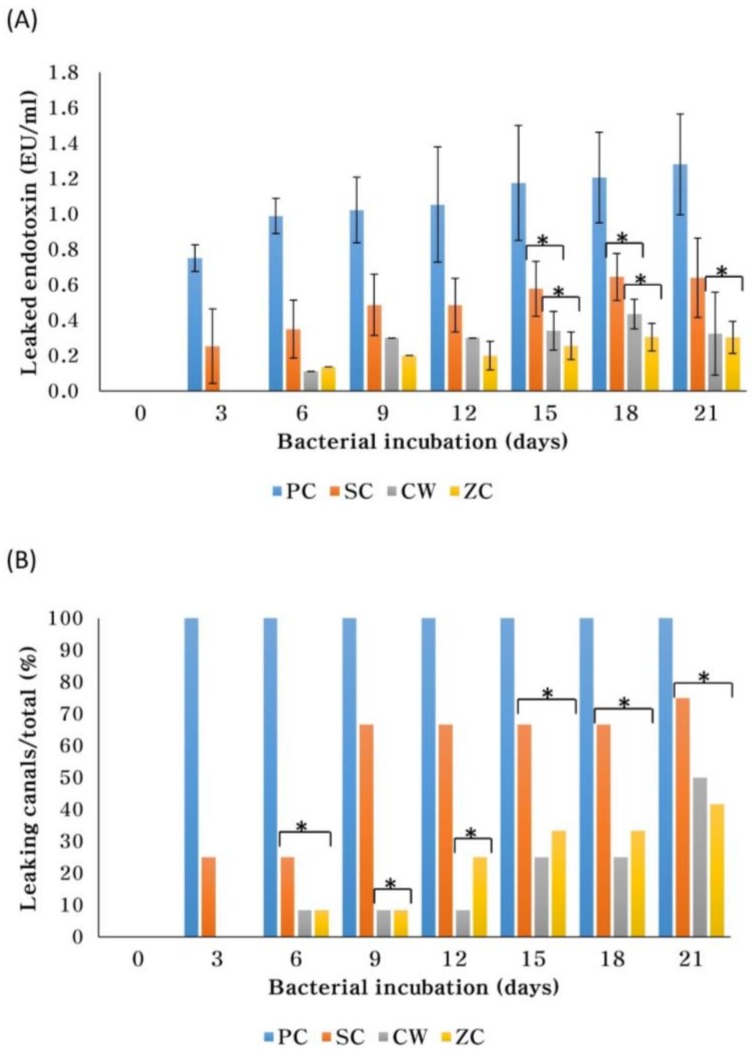
The amount (EU/mL) of endotoxin leakage (**A**) after 3, 6, 9, 12, 15, 18 and 21 days of bacterial incubation, and the ratio of root canals that leaked endotoxin (**B**). An asterisk (*) means no significant difference between groups. All negative control canals remained endotoxin-free for 21 days.

ZC-filled root canals showed a delay in endotoxin leakage (Table 1). All negative control canals remained endotoxin-free for 21 days, while all positive controls leaked endotoxin within 3 days (Figure 2). Several SC canals (25%) leaked in 3 days and most (67%) leaked within 9 days. However, only one canal in each of CW and ZC leaked after 6 days. Eventually, most SC canals (75%), half of CW (50%) and less than half of ZC (42%) leaked after 21 days. There were more SC canals than CW or ZC that leaked on the 3rd, 9th and 12th days. After 21 days, there was significantly more leaked endotoxin in the SC canals when compared to the leakage in the CW (*p* = 0.031) or ZC (*p* = 0.03) canals (Figure 2A). However, there was no significant (*p* > 0.05) difference in the amount of leaked endotoxin between the CW and ZC canals. 

**Figure 3:**
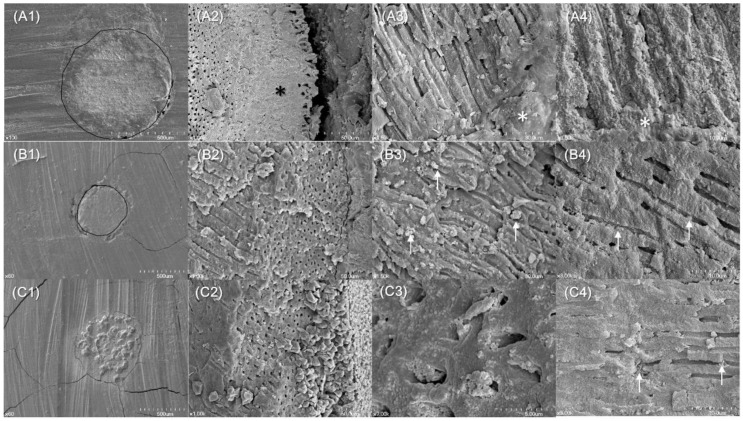
Scanning electron microscopic images of gutta-percha and AH 26 sealer (**A**), gutta-percha and EndoSequence BC sealer (**B**) and CaZrO_3_-containing calcium silicate cements (**C**) filled root canals of human premolars.

Biomineralization occurred in the ZC-filled dentinal tubules. The FE-SEM examination showed that CW canals had clear demarcations at the dentin–sealer interface (Figure 3(A1)) when the unsealed dentinal tubules (Figure 3(A2)) and the tubules covered with AH 26 sealer (Dentsply De Trey, Konstanz, Germany) were used (Figure 3(A3,4)). The SC canals showed partial separation of the EBC sealer from dentin and sealer impregnation of the dentinal tubules (Figure 3(B1,3,4)). The cross-sectional examination of ZC canals revealed dense fillings (Figure 3(C1)). ZC had penetrated into dentinal tubules and formed tag-like, rod-like and globular mineral structures (Figure 3(C3,4)).

(A1) (×100) A cross-sectional view at 2 mm from the root tip with GP demarcated by a circle. (A2) (×1000) Dentinal tubules on the root canal wall had some open orifices and other orifices that were partially blocked with the sealer (asterisk). (A3) (×1500) Some of the dentin on the canal walls was covered with AH 26 sealer (asterisk). (A4) (×3000) Orifices of dentinal tubules that were blocked with AH 26 sealer (asterisk).

(B1) (×60) A cross-sectional view at 2 mm from the root tip with GP demarcated by a circle. Distinguishing the EBC sealer from dentin was difficult due to the impregnation of dentin with a sealer, which is seen in the lower part of this image. There was a gap at the interface of dentine and filling material in the left portion of this image. (B2) (×1000) There were dentinal tubules with open orifices on the canal walls. (B3) (×1500) and (B4) (×3000) Dentin on canal walls was partially occluded with EBC sealer (arrows).

(C1) (×60) A cross-sectional view at 2 mm from the root tip, with the CaZrO_3_-containing calcium silicate cement shown to have densely filled the canal. (C2) (×1000) Most of the dentinal tubules orifices on the canal wall were covered with cubic-shaped crystals. (C3) (×7000) Tubule orifices were filled with tag-like structures that were partially fused with peritubular dentin. (C4) (×3000) There were rod-like mineralized structures (arrows) within the dentinal tubule.

## 3. Discussion

This study evaluated the sealing ability of different obturation materials and techniques by measuring endotoxin leakage with the LAL assay. The leakage models that use dyes, glucose, bacteria or radioisotopes may lack reproducibility, standardization and correlation between models [27]. Alternatively, the fluid filtration model is quantitative and allows repeated measurements, although only continuous coronal–apical voids are detectable [2]. The bacterial lipopolysaccharide is an endotoxin and is a general virulence factor present in the outer membrane of gram-negative bacteria that is predominantly involved in root canal infections [28]. The presence of endotoxin in infected root canals has a dose-dependent association with clinical and radiographic features of periapical disease [28,29]. Infected canals commonly contain *Porphyromonas gingivalis* (*P. gingivalis*), which were the bacteria used to measure leakage in this study [30]. Their endotoxin activates a proenzyme in the catalytic coagulation cascade of LAL in a dose-dependent manner and is thus measured in the LAL assay [31,32]. Additionally, limiting the assessment to straight mandibular premolars with single canals ensured better comparability [33]. 

The SC canals had more leakage than CW or ZC canals. SC canals required more sealer than the CW due to the size discrepancy between a single #40/06 GP cone and the canal, which particularly occurs within the middle and coronal thirds [34]. The larger volume of sealer around a single GP failed to provide a tight seal. Without compaction, there is little adaptation of GP to anatomic variations within canals and thus, a large volume of sealer may be unevenly distributed upon injection and result in voids [35]. The subsequent dissolution of the sealer causes leakage. Indeed, the EBC sealer is more soluble than either the epoxy resin- or calcium hydroxide-based sealers [12], although their solubility (2.9 ± 0.5%) meets the American National Standards Institute/American Dental Association requirements (<3%). 

ZC canals were as resistant to endotoxin leakage as CW. ZC hydration products may have contributed to its sealing ability according to the following theory. CaO may have reacted with water to form Ca(OH)_2_, which subsequently dissociated into Ca^2+^ and OH^−^ [36]. Ca^2+^ reacted with CO_2_ in tissues to form CaCO_3_ [36], which reduced gaps and increased retention [6]. Another hydration product that may have contributed to the seal is calcium silicate hydrate gel [37]. The gel forms tag-like structures in dentinal tubules and reacts with PO_4_^2−^ in dentinal fluid to form hydroxyapatite crystals that gradually occlude the tubules [15].

None of the obturating materials or techniques tested could completely seal canals. ZC microleakage may have increased the porosity of the cement [38]. Hydrated ZC has been shown to have porosities and microchannels due to insufficient packing, inadequate water/powder ratio or evaporation [17,38,39]. For calcium silicate cements, micro-CT (computed tomography) studies showed voids within the filling material and against the canal wall [39,40]. CW canal leakage was consistent with prior studies [34,41] and may have originated from inadequate bonding between the epoxy resin sealer and GP or dentin [2,6]. Some SC canals leaked as early as 3 days and many more leaked within 9 days before plateauing as the setting expansion of the EBC sealer reduced leakage [42]. Its setting was probably delayed in the humidifier, since prior studies found greater water absorption of calcium silicate cement [43] and faster settings when immersed in water [12,42,44]. 

There were differences in the sealer occlusion of dentinal tubules on the canal walls. In the CW canals, the AH 26 epoxy resin sealer formed a physical barrier without integrating with dentin (Figure 3(A3,4)). In contrast, the SC (EBC) and ZC canals showed actual penetration of dentinal tubules (Figure 3(B3,4) and Figure 3(C3,4)). Similarly, previous studies observed biomineralization by the calcium silicate cement at the cement–dentin interface [7,15]. However, in the current study, EBC composed of tricalcium silicate and calcium phosphate hybrids failed to form mineralized precipitates (Figure 3B), since calcium hydroxide was not produced by its hydration [23]. In contrast, ZC canals contained the cement and hydration products within their dentinal tubules. These hydration products were rod-like elongated structures (Figure 3(C3,4)) that could have blocked endotoxin penetration. However, despite differences in the penetration depth of obturating materials in the CW and ZC canals, their mean amounts of leaked endotoxin were comparable. Similarly, a previous study reported a lack of correlation between sealer penetration into dentinal tubules and the sealing ability of the sealer [45]. Since the endotoxin leakage model and micro-CT assessment are non-destructive assessment methods, further micro-CT evaluation of the tested specimens would be valuable [39,40].

Similarly, other studies have attested to the benefits of the calcium silicate cements with zirconium [20,21]. Calcium silicate cement with ZrO_2_ had more compressive strength and resulted in less inflammation in rat tissues than those with Bi_2_O_3_ [21]. Calcium silicate cement with 30% ZrO_2_ had setting times, an alkaline pH and calcium release that was similar to MTA [20]. However, since ZrO_2_ could hinder cement hydration with a heterogeneous phase [22], ZC was synthesized with calcium zirconate and tricalcium silicate (for radiopacity) to exclude the ZrO_2_ isolated previously. This synthesized calcium zirconate was scattered around tricalcium silicate particles (Figure 1). Canals filled with the novel ZC and canals obturated by CW resisted endotoxin leakage. Therefore, additional studies examining the push-out bond strength, flow, radiopacity, dimensional stability and a molecular analysis of the ZC hydration products are warranted. 

## 4. Materials and Methods

### 4.1. Synthesis and Morphology Analysis of Calcium Zirconate Containing Calcium Silicate

ZC was synthesized by a solid-state reaction using SiO_2_, CaO and ZrO_2_. The morphology of the synthesized powder was observed by FE-SEM (Model S4700) and STEM (Model JEM-2100F) equipped with EDS. The phase was examined by X-ray diffraction (Model D8-Advance) and compared with that of the synthesized tricalcium silicate cement without ZrO_2_, RetroMTA (BioMTA), and OrthoMTA (BioMTA).

### 4.2. Root Canal Preparation

Study approval was obtained from the Institutional Review Board at Seoul National University Dental Hospital, Seoul, Republic of Korea (ERI 16003). Extracted human mandibular premolars (*N* = 57) with straight single canals without previous root canal treatment, fractures below the cemento-enamel junction, root caries, root resorption or open apices were used. Periapical radiography was taken at the buccolingual and mesiodistal views to confirm a straight single root canal. After extraction, soft-tissue remnants were removed from teeth with thymol-soaked gauze. After this, the teeth were stored in 0.1% thymol at 25 °C for less than six months. Crowns were removed with a high-speed diamond (TR-13, Mani, Tochigi, Japan) and root lengths were standardized (12 ± 0.5 mm). The working length was established to be 1 mm from the apex, while canals were enlarged with ProTaper Next (Dentsply Maillefer, Ballaigues, Switzerland) up to X4 (#40/06). They were irrigated with 2 mL of 3.5% NaOCl between instruments, before being rinsed with 5 mL of 17% EDTA followed by 10 mL of 3.5% NaOCl to remove smear layer. Finally, they were flushed with 10 mL of saline to remove any remaining irrigant, before drying with paper points. The roots were randomly divided into three experimental (15 roots per group) and two control groups (6 roots per group). All canals were prepared and obturated by one endodontist.

### 4.3. Obturations

CW (*N* = 15): Root canals were obturated by the continuous wave of condensation technique using a #40/06 GP cone (Diadent, Chung-ju, Korea) and epoxy resin sealer (AH 26). Sealer was first coated onto GP and applied to the canal by a pumping motion. A B&L alpha II tip (B&L Biotech, Ansan, Korea) was activated and heated to 200 °C as it was inserted into the GP cone in the canal with light apical pressure until it reached a point that was 4–5 mm from the apical foramen. After this, the tip was deactivated for about 8s and again reactivated to remove the coronal portion of the master cone. The gutta-percha that remained within the apical portion was immediately compacted with a BL S-Kondenser (B&L Biotech). Finally, the coronal portion of the canal was obturated with thermo-plasticized gutta-percha (B&L-beta; B&L Biotech), before being vertically condensed with BL S-Kondenser. 

SC (*N* = 15): Root canals were obturated by a single cone technique using a #40/06 GP cone and EBC sealer^®^. EBC sealer was first dispensed into the canal through its syringe tip and a GP cone was inserted to the working length, according to the manufacturer’s instructions. A B&L alpha II tip was placed at the orifice of the canal, which was then activated and moved laterally without apical pressure to remove excess GP.

ZC (*N* = 15): Canals were obturated with ZC as previously described [15]. ZC powder was mixed into paste with distilled water and dispensed into the canal by OrthoMTA carrier (BioMTA). After this, the powder was plugged and spread using an OrthoMTA compactor file (BioMTA). The remaining canal was repeatedly filled with the ZC paste using BL-S Kondensers (B&L Biotech). Root canal obturating materials used in the present study are described in Table 1.

PC (*N* = 6): As positive controls, canals were obturated with GP without a sealer. 

NC (*N* = 6): As negative controls, unfilled canals had the entire root surface covered with nail varnish and sticky wax.

After obturation, all specimens were stored at 37 °C and 100% humidity for 1 week to ensure complete setting of the materials. A pilot study had revealed that ZC requires moisture and takes about 90 min to adequately set. After this, all root surfaces (except NC) were covered with nail varnish and sticky wax, leaving only the canal orifice and the apical 1 mm of the root exposed. 

### 4.4. Endotoxin Leakage

Leakage was measured through a dual chamber model as previously described [41,46]. The obturated (12/group) and control (6/group) roots were inserted into plastic 2-mL Eppendorf tubes with their tapered base removed. The gaps between the tube and root were sealed with acrylic resin and cyanoacrylate glue. All specimens were sterilized with ethylene oxide at 56 °C for four hours. Each root tip was submerged in 3 mL of Hank’s Balanced Salt Solution in a sterile flask, which was then sealed with acrylic resin and cyanoacrylate glue. One-mL aliquots of *P. gingivalis* were placed on the coronal root at the Eppendorf tube entrance, which were then incubated in an anaerobic chamber at 37 °C for 21 days. Fresh bacterial suspensions were added every three days. The lower chamber of the sterile flask was sampled every third day for 21 days. The *P. gingivalis* endotoxin was measured using the LAL assay (Lonza, Walkersville, MD, USA).

### 4.5. Statistical Analysis

Leaked endotoxin concentrations and ratios of leaked specimens were analyzed by one-way ANOVA with Scheffe’s test (*α* = 0.05) using SPSS statistics version 20 (IBM, Armonk, NY, USA). 

### 4.6. Biomineralization Identified

Ultrastructural features were examined by FE-SEM (CW/SC/ZC, *n* = 3/group). These obturated roots were stored in an incubator with 100% humidity at 37 °C for 21 days, before being cross-sectioned at 2 mm from the root tip with a low speed diamond disk (Kerr, Orange, CA, USA). The coronal segments were split longitudinally through the center by creating longitudinal grooves on their outer surfaces with a low-speed diamond disk, before applying a blade and hammer. These cross-sections and segments were mounted on aluminum stubs and sputter-coated with a 30-nm layer of gold. The cross-sectional surfaces and filling-dentin interface were examined by FE-SEM (Model S 4700) at ×60, 100, 1000, 1500, 3000 and 7000 magnifications. 

## 5. Conclusions

Canals filled with a novel CaZrO_3_-containing calcium silicate cement alone were as resistant to endotoxin leakage as those obturated by CW with GP and AH 26 sealer; and much more resistant than a single cone obturation with GP and EBC sealer. Canals sealed with this ZC cement had mineralization within dentinal tubules over 21 days. The novel CaZrO_3_-containing calcium silicate cement may be suitable to be used as a canal obturating material. 

## Figures and Tables

**Table 1:** The mean amount of endotoxin leakage (EU/mL) and ratio of the leaked specimens measured after 21 days of exposure for the experimental groups and control groups.

Group	NC	PC	CW	SC	ZC
Amount of leaked endotoxin (Mean ± SD) (EU/mL)	<0.01	1.281 ± 0.284	0.324 ± 0.235	0.641 ± 0.225	0.303 ± 0.091
(% of PC) *	(<1%)	(100%)	(25%)	(50%)	(24%)
Number leaked/total (%)	0/6 (0%)	6/6 (100%)	6/12 (50%)	9/12 (75%)	5/12 (42%)

NC: negative control; PC: positive control; CW: continuous wave of condensation with GP and AH 26 sealer; SC: single GP cone obturation with EndoSequence BC sealer; ZC: obturation with CaZrO_3_-containing calcium silicate cement. * Percent ratio of the amount of leaked endotoxin relative to the amount of leaked endotoxin in PC.
